# Screening for immune-potentiating antigens from hepatocellular carcinoma patients after radiofrequency ablation by serum proteomic analysis

**DOI:** 10.1186/s12885-018-4011-8

**Published:** 2018-01-31

**Authors:** Shunli Shen, Hong Peng, Ye Wang, Ming Xu, Manxia Lin, Xiaoyan Xie, Baogang Peng, Ming Kuang

**Affiliations:** 1 Department of Liver Surgery, The First Affiliated Hospital, Sun Yat-sen University, 58 Zhongshan Road 2, Guangzhou, 510080 People’s Republic of China; 2 Department of Bilio-pancreatic Surgery, The First Affiliated Hospital, Sun Yat-sen University, 58 Zhongshan Road 2, Guangzhou, 510080 People’s Republic of China; 3 Division of Interventional Ultrasound, The First Affiliated Hospital, Sun Yat-sen University, 58 Zhongshan Road 2, Guangzhou, 510080 People’s Republic of China; 4 Department of Medical Ultrasonics, The First Affiliated Hospital, Sun Yat-sen University, 58 Zhongshan Road 2, Guangzhou, 510080 People’s Republic of China

**Keywords:** Hepatocellular carcinoma, Radiofrequency ablation, Proteomics, Ficolin-3, Immunotherapy

## Abstract

**Background:**

Radiofrequency ablation (RFA) can not only effectively kill hepatocellular carcinoma (HCC) tumour cells but also release tumour antigens that can provoke an immune response. However, there is no consensus regarding which antigens could constitutively be generated after RFA and could potentiate the immune response. The aim of this study was to identify these immune-potentiating antigens.

**Methods:**

We performed two-dimensional electrophoresis (2-DE) and MALDI-TOF-MS/MS analyses on serum obtained before and after RFA from 5 HCC patients. Further validation for selected proteins was performed utilizing ELISA analysis on another 52 HCC patients. Disease-free survival (DFS) analysis according to the differential expression of the interested protein before and after RFA was performed.

**Results:**

Twelve decreased and 6 increased proteins after RFA were identified by MS. Three proteins, including clusterin, Ficolin-3, and serum retinol binding protein-4, were further verified by ELISA on the 52 HCC patients. Only Ficolin-3 proved to be significantly changed after RFA. The 52 patients were divided into two groups according to the expression of Ficolin-3 before and after RFA. The 1-, 2- and 3-year DFS rates were 59.1%, 31.8%, and 22.7%, respectively, for patients in the low Ficolin-3 group (22 patients) and 73.3%, 60.0%, and 50.0%, respectively, for patients in the high Ficolin-3 group (30 patients) (*P* = 0.038).

**Conclusions:**

In conclusion, Ficolin-3 was overexpressed in the serum of most HCC patients after RFA. Ficolin-3 might be a biomarker for RFA treatment efficacy and a potential target for HCC immunotherapy.

## Background

Hepatocellular carcinoma (HCC) is the fifth most common malignancy and the third leading cause of cancer-related deaths worldwide, occurring most frequently in the setting of chronic liver injury and cirrhosis. Despite improvements in the diagnostic and treatment modalities, the long-term prognosis has been far from satisfactory [1, 2].

Radiofrequency ablation (RFA) has been established as the primary ablative modality for HCC. However, similar to other treatments, RFA for HCC is frequently followed by tumour recurrence [3]. There is an urgent need to develop novel therapies with systemic activity to avoid spontaneous progression or recurrence of HCC. Immunotherapy represents a potential therapeutic option.

It has been shown that RFA can not only effectively kill HCC tumour cells but also provoke the immune response to remove the debris [4, 5]. At temperatures above 45 °C to 50 °C, cell membranes are destroyed, proteins are denatured, and a region of necrosis surrounding the electrode is generated [6]. Hyperthermia-related tumour antigenicity suggests that thermal ablation-inactivated tumour tissues are endowed with immunogenicity, which might be closely related to the necrotic tumour cell-released molecules with high antigenic characteristics. In addition, the inflammatory response resulting from tumour necrosis following thermal ablation may lead to the accumulation of a large number of antigen-presenting cells (APCs) and lymphocytes at the treatment sites. These necrotic tumour cell-activated APCs, which are more dynamic than those activated by apoptotic cells, can more effectively promote the effect of antigen presentation to effector cells [7, 8].

Therefore, RFA favours immune activation and the presentation of otherwise cryptic antigens, thus inducing a specific anti-tumour immune response. However, unfortunately, there is no consensus regarding which antigens could constitutively be generated after RFA and could potentiate the immune response.

To identify the immune-potentiating antigens, proteomic analysis with two-dimensional electrophoresis (2-DE) and mass spectrometric analysis (MS) was used in this study. We found that Ficolin-3 was overexpressed in the serum of most HCC patients after RFA. In addition, elevated Ficolin-3 predicts better prognosis, which might be a biomarker for RFA treatment efficacy and a potential therapeutic target for HCC immunotherapy.

## Methods

### HCC patients

This study was performed according to the guidelines of the Helsinki Declaration. It was registered and approved by the ethics committee at The First Affiliated Hospital of Sun Yat-sen University. All patients provided written informed consent before treatment. The serum samples were collected from 57 primary HCC patients who underwent RFA treatment at the First Affiliated Hospital of Sun Yat-sen University between January 2012 and March 2013. All patients underwent abdominal ultrasound and computed tomography (CT) or magnetic resonance imaging (MRI), chest X-ray or CT before RFA. All of these patients were more than 18 years of age with complete clinical and laboratory data. Patients who received preoperative chemotherapy or radiotherapy were excluded. In addition, no patient had coexistent haematologic disorders or known active infection before treatment, ensuring that the serum parameters tested were representative of the normal baseline value. Specimens were obtained with informed consent from all patients. Sera were collected before RFA and 7 days after RFA from each patient. Fine needle biopsy was carried out on each patient before RFA. All patients were histologically diagnosed with HCC. Patients with mixed HCC and cholangiocarcinoma or without full follow-up data were excluded.

### Sample preparation for proteomic analysis

The blood samples of 5 of the 57 HCC patients were collected into clean glass tubes without an additive and were allowed to clot at room temperature for 60 min. Serum was separated by centrifugation at 1000 x *g* for 30 min to remove the insoluble solids. Aliquots of serum were then stored at − 80 °C until use. The removal of albumin and IgG was performed using the ProteoPrep Blue Albumin Depletion kit (Sigma, St. Louis, MO, USA), according to the manufacturer’s instructions. The 2-D cleanup kit (GE Healthcare, UK) was used to remove impurities from the protein extraction prior to the determination of the sample concentration using the 2-D Quant kit (GE Healthcare).

### 2-de

Proteins derived from 5 samples before and after RFA were pooled separately, and 2-DE was performed three times per sample to minimize gel-to-gel variations. The Immobiline Dry strip (pH 4–7 L, length 18 cm; GE Healthcare) was immersed with 120 μg of proteins in 350 μl of rehydration buffer containing 5 M urea, 1 M thiourea, 4% CHAPS, 65 mM dithiothreitol, 5 mM tributylphosphine, 1% IPG buffer, and 1 mM phenylmethylsulphonyl fluoride. Isoelectric focusing (IEF) was performed using an IPGphor IEF apparatus with 0.002% bromophenol blue for 14 h at room temperature (GE Healthcare) at 70 kVh. The strip was then subjected to two-step equilibration in equilibration buffer containing 6 M urea, 30% glycerol, 2% SDS and 50 mM Tris-HCl (pH 6.8) with 2% dithiothreitol (*w*/*v*) for the first step and 2.5% (w/v) iodoacetamide for the second step. The two-dimensional SDS-PAGE gel (12.5% T, 18× 16 × 0.015 cm) was run at 7 W for 30 min followed by 17 W for 4 h. Separated proteins were stained with Deep Purple fluorescence dye (GE Healthcare; 1:200 diluted in 100 mM borate buffer) at room temperature for 1.5 h and then were rinsed 3 times (5 min each) with deionized water. The resolved protein spots in individual stained 2-D gels were visualized using a Typhoon 9200 laser scanner (GE Healthcare).

### In-gel enzymatic digestion

ImageMaster 2-D Elite software 5.0 (GE Healthcare) was used for image analysis, which included spot detection, quantification and normalization. The intensity volume of each spot was normalized with the total intensity volume (summation of the intensity volumes obtained from all spots within the same 2-D gel) and was expressed as the relative intensity. In-Gel Enzymatic Digestion Protein spots were excised from the gel with an Ettan Spot Picker (version 1.0, GE Healthcare), destained twice with 30 mM potassium ferricyanide and 100 mM sodium thiosulphate (1:1, *v*/v) and then equilibrated in 50 mM NH_4_HCO_3_ to pH 8.0. After dehydration with acetonitrile (ACN) and drying in a speed vacuum concentrator for 20 min, the gel pieces were rehydrated in a minimal volume of sequencing grade porcine trypsin (Promega) solution (20 μg/ml in 25 mM NH_4_HCO_3_) and were incubated at 37 °C overnight. The peptides were extracted twice using 0.1% TFA in 50% ACN and were completely dried in a speed vacuum concentrator.

### Protein identification and database searching

MALDI-TOF-MS/MS identification and database searching protein identification were performed using an Ultraflex III mass spectrometer (Bruker Daltonics, Bremen, Germany) operated in the reflectron mode at an accelerating voltage of 20 kV. A saturated solution of α-cyano-4-hydroxycinnamic acid in 50% ACN and 0.1% TFA was used as the matrix. Peptide mass fingerprints and MS/MS analysis were searched using BioTools software (version 3.0, Bruker Daltonics, Germany) against the SwissProt protein database. Protein identification was accepted when the peptide score was higher than the threshold value (*P* < 0.05), and manual interpretation had to confirm the agreement between the spectra and peptide sequence.

### Enzyme-linked immunosorbent assay (ELISA) analysis

The levels of Clusterin (CLU) (E91180Hu; Cloud Clone Co.), Ficolin-3 (FCN3) (E91903Hu; USCN), and retinol binding protein 4 (RBP4) (E90929Hu; Cloud Clone Co.) in serum were measured using ELISA according to the manufacturer’s instructions. After development with a chromogen-substrate solution, the reaction was terminated by adding 100 μl of stop solution. Optical density values were read at 450 nm, and the concentrations were automatically calculated according to the standard curve.

### Follow-up

Patients were regularly followed up at outpatient clinics every month for the first half year, every 3 months for the next one and a half years, and once annually thereafter. Patients received a physical examination, liver ultrasound, chest X-ray and serum alpha foetal protein (AFP) test at each follow-up. Abdominal computed tomography (CT) was performed every 6–12 months or when recurrence was suspected. Recurrence was defined as the emergence of clinical, radiological, and/or pathologic diagnosis of tumours from a previous origin locally or distantly. Once recurrence was confirmed, salvage treatments, including percutaneous ablation, surgery, or transcatheter arterial chemoembolization (TACE) were selected as needed.

### Statistical analysis

Statistical analysis was performed using SPSS statistical software (SPSS Inc., Chicago, IL, USA, version 16.0 for Windows). Student’s *t*-test and one-way analysis of variance (ANOVA) were used to analyse differences between groups. Disease-free survival (DFS) was calculated from the date of RFA to the date of recurrence. Survival curves were plotted using the Kaplan-Meier method and were compared using the log-rank test. A *P*-value < 0.05 was considered statistically significant.

## Results

### Patient and tumour characteristics

There were 49 (86.0%) male and 8 (14%) female patients. The mean age of the patients was 55.8 ± 12.6 years (range: 30-76 years). Hepatitis B surface antigen (HBsAg) was positive in 50 patients (87.7%). Increased AFP (> 20 ng/ml) was found in 34 cases (59.6%). Sixteen (28.1%) patients had more than one tumour in the liver with a mean tumour size (greatest dimension) of 2.7 ± 0.8 cm (range: 1-4.4 cm). Thirty-two patients (56.1%) developed recurrence and 21 (36.8%) died during follow-up.

### Proteomic analyses identified differentially expressed serum proteins after RFA

**Fig. 1:**
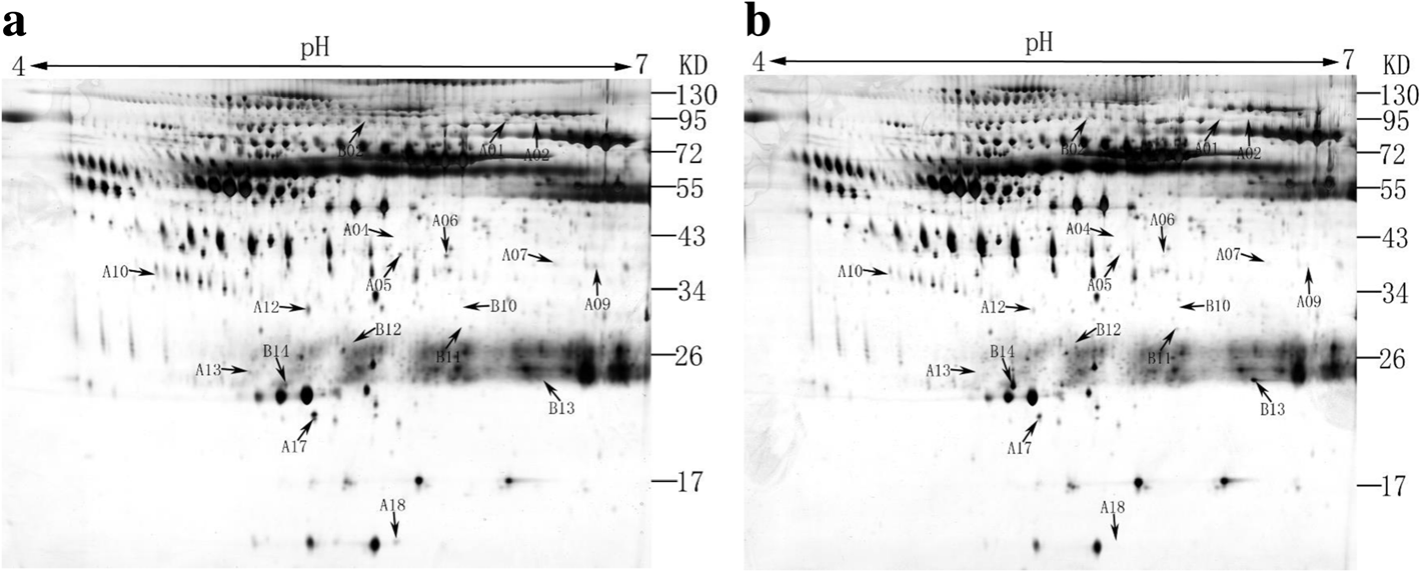
Representative 2-DE serum protein profiles before and after RFA treatment for hepatocellular carcinoma patients. These differentially expressed protein spots (labelled as A1 to A18 and B2 to B14) were subsequently identified by MALDI-TOF MS/MS analyses

**Fig. 2:**
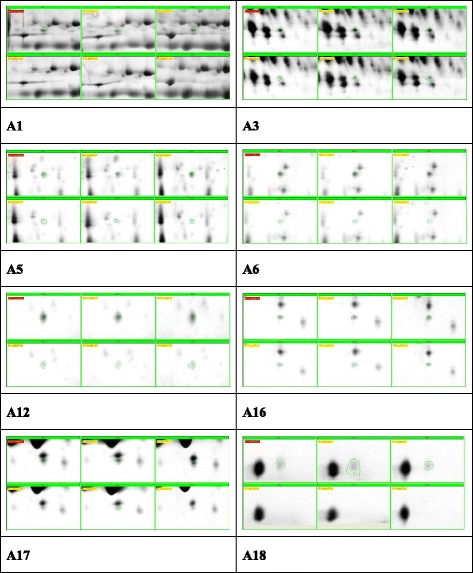
Protein spots decreased after RFA

**Fig. 3:**
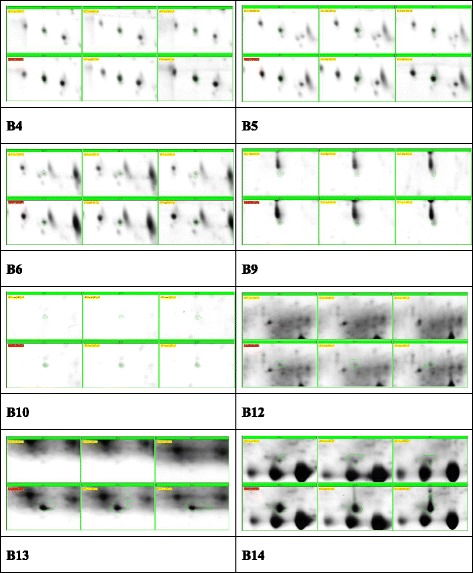
Protein spots increased after RFA

Representative gel images of the protein are shown in Fig. 1. A comparison of the 2-DE images revealed that 18 protein spots (A1-A18) were down-regulated (Fig. 2) and 16 protein spots (B1-B16) were up-regulated after RFA (Fig. 3), in which 12 and 6 protein spots changed more than two-fold, respectively. We next identified these 18 differentially expressed host-specific proteins by mass spectrometry, including 12 proteins that were decreased after RFA and 6 proteins that were increased after RFA treatment (Table 1). Finally, three of these candidates were found to be possibly immunoreactive through document review: CLU, Ficolin-3 and RBP4 [9, 10].

**Table 1:** Protein identification using MALDI-TOF MS/MS

No.	gi|	Protein name
A01	251,837,060	Chain D, Cobra Venom Factor (Cvf) In Complex With Human Factor B
A02	110,590,599	Chain A, Apo-Human Serum Transferrin (Glycosylated)
A04	78,174,390	HP protein
A05	220,702,427	Chain B, Crystal Structure Of Bbetad432a Variant Fibrinogen Fragment D With The Peptide Ligand Gly-His-Arg-Pro-Amide
A06	7,770,217	PRO2675
A07	49,258,810	Chain A, Human Serum Transferrin, N-Lobe Bound With Oxalate
A09	158,254,550	unnamed protein product
A10	193,787,502	unnamed protein product
A12	32,891,795	clusterin (complement lysis inhibitor, SP-40,40, sulfated glycoprotein 2, testosterone-repressed prostate message 2, apolipoprotein J)
A13	86,439,006	immunoglobulin lambda light chain
A17	7,770,173	PRO2222 (RBP4)
A18	212,374,952	Chain A, Crystal Structure Of Transthyretin Variant V20 s
B02	2,781,209	Chain C, Crystal Structure Of Fibrinogen Fragment D
B10	3,413,516	Hakata antigen (H-ficolin)
B11	119,626,073	albumin, isoform CRA_j
B12	576,259	Chain A, The Structure Of Pentameric Human Serum Amyloid P Component
B13	149,673,887	immunoglobulin light chain
B14	170,684,606	immunoglobulin kappa 1 light chain

### ELISA analysis

**Fig. 4:**
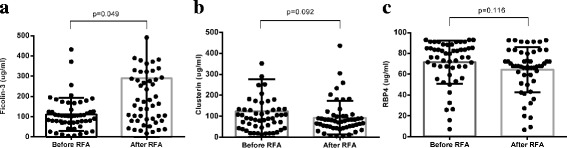
Ficolin-3, CLU and RBP4 levels in serum before and after radiofrequency (RFA) was compared in a validation set of 52 patients using ELISA. Results are expressed as the mean±SD. A *P*-value <0.05 was considered statistically significant

We further expanded our sample size and validated the differential expression of CLU, Ficolin-3 and RBP4 in sera from another 52 samples using ELISA. The mean values of CLU, Ficolin-3 and RBP4 before and after RFA were 121.9 ± 21.5 Vs 92.9 + 11.1 μg/ml (range: 5.008 to 1087.0 μg/ml), 110.4 + 11.4 Vs 289.7 + 87.4 (range: 4.34 to 4630 μg/ml), and 71.6 + 2.9 Vs 64.4 + 3.0 (range: 6.6 to 93 μg/ml), respectively. Through comparison of the serum levels of the above 3 proteins before and after RFA, only Ficolin-3 showed a significant difference (*P* < 0.05) (Fig. 4).

### Survival analysis

**Fig. 5:**
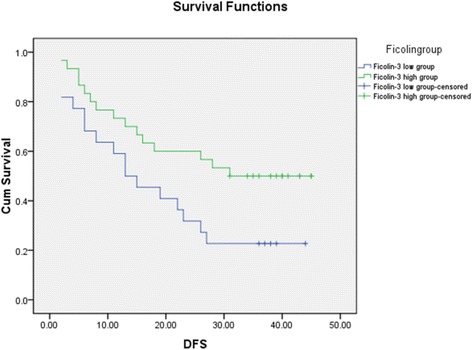
Disease-free survival of HCC patients after RFA according to the expression level of Ficolin-3

Fifty-two patients were divided into two groups according to the expression mode of Ficolin-3. Thirty patients with elevated Ficolin-3 after RFA were divided into the high Ficolin-3 group, while 22 patients with decreased Ficolin-3 after RFA were divided into the low Ficolin-3 group. Basic clinical characteristics including gender, age, Child-Pugh staging, AFP level, tumour size and tumour number between the two groups showed no significant difference (P < 0.05). The 1-, 2- and 3-year DFS rates were 59.1%, 31.8%, and 22.7% for patients in the low Ficolin-3 group and 73.3%, 60.0%, and 50.0% for patients in the high Ficolin-3 group. (*P* = 0.038) (Fig. 5).

## Discussion

RFA is a minimally invasive technique used as standard local therapy for HCC. Its immune-potentiating effect has been frequently reported with few studies concentrating on the exact mechanisms. In the present study, we performed a gel-based serum proteomic analysis before and after RFA for HCC patients and identified 18 proteins to be differentially expressed by mass spectrometry. Through literature review, we found three proteins, CLU, Ficolin-3, and RBP4, that might be related to an immune response. Further ELISA assay showed that only Ficolin-3 significantly changed after RFA. Survival analysis showed that patients with higher Ficolin-3 after RFA tended to have better DFS, indicating that Ficolin-3 might be one of the immune-potentiating tumour antigens.

The exact explanation for the association between the enhanced immune response and RFA is not fully clarified. There are several possible explanations. First, RFA destroyed the tumour and relieved the body of the tumour burden, possibly leading to the reversal of immune suppression and unmasking a population of primed tumour-specific T cells that can mediate protective immunity [11]. Second, RFA leads to enhanced release and exposure of immune-potentiating tumour antigens, such as nuclear protein high mobility group B1 and heat shock proteins (HSPs), which might induce the antitumour immune response through the activation of dendritic cells (DCs) [12]. At 24 h after percutaneous treatment of HCC by thermal ablation, the expression of HSP70 in the cytoplasm and on the cell membrane of tumour cells was augmented by 8 times and that of HSP90 was augmented by 1.2 times. The two HSPs are evidently antigenic stimulatory to the immune system [13]. The anti-tumour effect of in vitro-inactivated vaccine is comparatively weak, which might be due to the rinsing of many immunogens (such as HSP) released by necrotic tumour cells during multiple centrifugal washing processes, resulting in immunogenicity decline of the tumour cells finally collected. This indirectly proves the effectiveness of the anti-tumour immune response stimulated by tumour-associated antigens released in situ from tumour tissues following thermal ablation. Ficolin-3 might be one of these in situ-released antigens. Third, hyperthermia induced by RFA causes both immunologic and biologic effects, such as the accelerated migration of peripheral blood mononuclear cells, activation of effector cells, and induction and secretion of cytokines, all of which might enhance the antitumour immune response [14]. Hyperthermia-killed HCC cells express a large number of HSPs. Compared with tumour cells untreated with heat shock, hyperthermia-treated ones have sensitized dendritic cell vaccines more effective in inducing CD4^+^ and CD8^+^ T cells to participate in the anti-tumour immune response [15]. In addition, HCC ablation induces a functional transient activation of myeloid dendritic cells (MDC) associated with increased serum levels of TNF-α and IL-1β [16].

We found that serum Ficolin-3 was significantly overexpressed after RFA, which was verified by ELISA in 52 specimens. Furthermore, we found that patients with higher Ficolin-3 after RFA had better 1-, 3-, and 5- year DFS. However, no significant difference was found for CLU and RBP4. Ficolin-3 (H-ficolin; Hakata antigen) is a thermolabile beta-2-macroglycoprotein found in all human sera and is a member of the ficolin/opsonin p35 lectin family. The protein can activate the complement pathway in association with MASPs and sMAP, thereby aiding in the host defence through the activation of the lectin pathway [17]. It was found that a low concentration of Ficolin-3 was associated with an increased risk of fever and neutropenia (FN), particularly FN with bacteraemia, in children treated with chemotherapy for cancer [18]. In addition, Ficolin-3 was found to bind to IgMs, and the IgM-Ficolin-3 complex deposited on cancer cells could induce complement attack. Therefore, the IgM-Ficolin-3 -mediated complement activation pathway might be a new defensive strategy for human cancer immunosurveillance. These two studies showed that high Ficolin-3 represents strong immunity against infection or cancer. We proposed this might be one of the reasons for better survival for HCC patients with higher serum Ficolin-3 after RFA.

Because the immune-potentiating effect of thermal ablation is not sufficiently strong to prevent tumour recurrence, there is an urgent need to develop novel therapies with systemic activity to avoid spontaneous progression or recurrence after RFA treatment [19]. In a rabbit VX2 hepatoma model, it was found that combined RFA and Toll-like receptor 9 (TLR9) agonist stimulation not only induced increased antitumour T-cell stimulation/cytotoxicity and a longer mean survival of animals but also significantly inhibited tumour spread to the lungs and peritoneum and prohibited new tumour growth in animals receiving a secondary systemic tumour cell injection [20]. In HCC patients, Cui et al. found that cellular immunotherapy (CIT) with autologous mononuclear cell-derived natural killer (NK) cells, γδT cells and cytokine-induced killer (CIK) cells could enhance progression-free survival for HCC patients after RFA with no adverse response [21]. den Brok et al. found that RFA induced a weak immune response, but it was not sufficient to protect against tumour recurrence. Therefore, they combined immune-potentiating strategies via the administration of CTLA-4-blocking antibodies to lower the threshold for T-cell activation and obtained a better immune response, finally leading to increased tumour protection [22]. Actually, we conceived the same idea as den Brok that tumour antigens released by tumours in situ or in the serum might induce an antitumour immune response, leading to a better prognosis, and Ficolin-3 might be one of those antigens.

Our study possesses limitations. First, in this study, we tried to identify immune-potentiating tumour antigens released after RFA, but we could only find antigens soluble in serum instead of other tumour antigens in situ. It would have been more ideal to have used ablated tumour tissues in this study. However, in clinical practice, it is very difficult to obtain sufficient tumour antigens. This idea is undoubtedly possible in animal models, although it would not reflect the true circumstance. Second, most of the HCC patients were HBV associated in the present study. Thus, it remains to be verified in what type of model would Ficolin-3 change after RFA using other hepatitis-associated HCC samples. Additionally, although we found that Ficolin-3 might be used as a tumour vaccine for the prevention and therapy of residual tumour through stimulation of the immune system, we have not proven this hypothesis, which we hope to confirm in a future animal study or even with preclinical tests.

## Conclusions

We found that Ficolin-3 was overexpressed in the serum of most HCC patients after RFA and might be a potential biomarker for RFA treatment efficacy and tumour vaccine development for HCC immunotherapy. The combination of RFA and active immunotherapy with Ficolin-3 may have a synergistic effect in HCC treatment.

## Abbreviations

2D-DIGE: Two-dimensional difference gel electrophoresis
2-DE: Two-dimensional electrophoresis (2-DE)
ACN: Dehydrated with acetonitrile
AFP: Alpha foetal protein
ANOVA: One-way analysis of variance
APCs: Antigen-presenting cells (APCs)
CIK cell: Cytokine-induced killer cell
CIT: Cellular immunotherapy
CLU: Clusterin
DFS: Disease-free survival
ELISA: Enzyme-linked immunosorbent assay
FCN3: Ficolin-3
FN: Fever and neutropenia
HCC: Hepatocellular carcinoma
HMGB1: Nuclear protein high-mobility group B1
HSPs: Heat shock proteins
IEF: Isoelectric focusing
MDC: Myeloid dendritic cells
MS: Mass spectrometric analysis
NK cell: Natural killer cell
RBP4: Retinol binding protein 4
RFA: Radiofrequency ablation
TACE: Transcatheter arterial chemoembolization
TLR9: Toll-like receptor 9
